# Pressure-Natriuresis Response Is Diminished in Old Age

**DOI:** 10.3389/fcvm.2022.840840

**Published:** 2022-02-16

**Authors:** Yang Gyun Kim, Ju-Young Moon, Bermseok Oh, Ho Jun Chin, Dong Ki Kim, Jung Hwan Park, Sung Joon Shin, Bum Soon Choi, Chun Soo Lim, Sang Ho Lee

**Affiliations:** ^1^Division of Nephrology, Department of Internal Medicine, Kyung Hee University College of Medicine, Seoul, South Korea; ^2^Department of Biomedical Engineering, School of Medicine, Kyung Hee University, Seoul, South Korea; ^3^Division of Nephrology, Department of Internal Medicine, Seoul National University Bundang Hospital, Seongnam, South Korea; ^4^Division of Nephrology, Department of Internal Medicine, Seoul National University Hospital, Seoul, South Korea; ^5^Division of Nephrology, Department of Internal Medicine, Konkuk University School of Medicine, Seoul, South Korea; ^6^Division of Nephrology, Department of Internal Medicine, Dongguk University Ilsan Hospital, Goyang, South Korea; ^7^Division of Nephrology, Department of Internal Medicine, Eunpyeong St. Mary's Hospital, College of Medicine, The Catholic University of Korea, Seoul, South Korea; ^8^Division of Nephrology, Department of Internal Medicine, Seoul National University Boramae Medical Center, Seoul, South Korea

**Keywords:** salt sensitivity, pressure, natriuresis, hypertension, old

## Abstract

**Background:**

Age-related alterations in renal sodium handling affect blood pressure (BP). We aimed to clarify whether the pressure-natriuresis response changes with age, leading to BP elevation.

**Methods:**

A total of 4,859 participants with normal renal function from the Korean Genome and Epidemiology Study (KoGES) and 235 patients with non-diabetic chronic kidney disease (CKD) from the ESPECIAL trial were included and divided into the younger and older groups. In ESPECIAL, participants took olmesartan from weeks 0 to 16 and were educated about a low-salt diet (LSD) from weeks 8 to 16.

**Results:**

In both studies, older participants showed lower estimated glomerular filtration rate (eGFR) and urine concentration index and higher albuminuria. In KoGES, BP was higher and urine sodium was lower in older participants. In ESPECIAL, diastolic BP at 0 week was lower in older participants. Olmesartan reduced BP in both groups, whereas LSD decreased systolic BP only in older participants. Urine sodium increased in younger participants but decreased in older participants after olmesartan use. In KoGES, urine sodium was correlated with BP in both groups after adjusting for age, sex, and eGFR; however, the correlation coefficient was lower in older participants. In ESPECIAL, only younger participants showed a significant positive association between systolic BP and urine sodium in multiple regression analysis.

**Conclusions:**

The pressure-natriuresis response was diminished in older participants with or without CKD.

## Introduction

The prevalence of hypertension is reported to be >75% in adults aged >65 years, and hypertension-related complications also increase with age (1). Despite the benefits of blood pressure (BP) lowering, <50% of older adults achieve the guideline-recommended BP target (2, 3). The mechanisms underlying age-related BP elevation remain unclear. However, alterations in sodium homeostasis with age have been shown to result in salt retention and consequently to hypertension in animal and human studies. The kidney undergoes structural and functional changes with advancing age, including reduction in renal blood flow and glomerular filtration rate (GFR) (4). In animal studies, old rats were observed to excrete less sodium during acute volume expansion or in response to angiotensin II (ANGII) infusion than young rats (5, 6). Aged mice also showed lower sodium excretion compared with young mice at a similar BP (7). Similarly, in human studies, participants older than 40 years excreted less sodium after normal saline loading than those younger than 40 years (8). The average BP increased with age, and the incidence of salt-sensitive hypertension was higher in individuals with advanced age than in those with younger age (9). Pressure natriuresis is a crucial factor that controls extracellular volume and ultimately regulates BP. Therefore, age-related decline in sodium excretion and a shift to the right of the pressure-natriuresis curve may be the main causes of increased salt-sensitive hypertension in the older population.

However, the decline in the natriuretic response is not simply explained by reduced GFR. Several studies have identified that urine sodium levels are not associated with estimated GFR (eGFR) (10, 11). Senescent rats showed blunted natriuresis even with high degrees of renal perfusion pressure and normal BP (12). Increased renal sympathetic tone and decreased natriuretic peptide levels were suggested as factors that inhibit renal sodium excretion (6, 13). However, large-scale human data demonstrating changes in age-related sodium handling are lacking. We hypothesized that the pressure-natriuresis response may be diminished in old age regardless of the renal function status. In this study, we aimed to clarify the difference in pressure natriuresis between younger and older age groups by using data from two different studies conducted in participants with normal kidney function and patients with non-diabetic chronic kidney disease (CKD).

## Materials and Methods

### Study Design and Participants

First, data were obtained from the Korean Genome and Epidemiology Study (KoGES), a prospective, longitudinal study (14). The KoGES recruited community residents from Ansan (urban area) and Ansung (rural area), South Korea. Eligible participants aged between 40 and 69 years were voluntarily enrolled at baseline. Initially, 10,030 individuals participated in the baseline surveys and physical examinations in 2001–2002. All participants provided informed consent for undergoing laboratory tests and an interview survey at baseline. BP was measured in sitting position at the baseline visit following relaxing time for at least 10 minutes at the left arm by the auscultatory method (15). Of the original cohort (*n* = 10,030), 4,936 participants who provided urine samples were included. Further, we excluded individuals with eGFR calculated using the Modification of Diet in Renal Disease (MDRD) equation (MDRD eGFR) <60 mL/min/1.73 m^2^ (*n* = 69) and those using diuretics (*n* = 8). A total of 4,859 participants were included in the final analysis. This study was approved by our institutional review board (KHNMC 2020-01-019-002), and de-identified data from the KoGES were acquired under a data-sharing agreement with the Division of Genetic Epidemiology and Health Index.

Second, data were obtained from the ESPECIAL (Effects of Low Sodium Intake on the Antiproteinuric Efficacy of Olmesartan in Hypertensive Patients with Albuminuria) trial (clinicaltrials.gov registration no. NCT01552954) (16). A total of 235 patients with non-diabetic CKD (MDRD eGFR ≥ 30 mL/min/1.73 m^2^) and albuminuria (urine albumin/creatinine ratio ≥ 30 mg/g creatinine) were enrolled from seven renal clinics in Korea (Seoul National University Boramae Medical Center, Seoul National University Bundang Hospital, Seoul National University Hospital, Konkuk University Hospital, Dongguk University Ilsan Hospital, Kyung Hee University Medical Center, and Seoul St. Mary's Hospital) between 2012 and 2013. Their ages ranged from 19 to 75 years. Patients with uncontrolled hypertension (BP > 160/110 mmHg), hyperkalemia (potassium level > 5.5 mEq/L), malignant disease, cerebral vascular disease (cerebral infarction, hemorrhagic infarction, acute myocardial infarction or unstable angina, coronary angioplasty, or coronary artery bypass surgery) within 6 months, and diabetes mellitus or pregnancy were excluded. During the run-in period for 8 weeks, all renin–angiotensin–aldosterone system (RAAS) blockers or diuretics were stopped and changed to different antihypertensive agents (Supplementary Figure 1). Olmesartan (40 mg) was prescribed once daily for 16 weeks (from weeks 0 to 16). Two types of low-salt diet (LSD) education (intensive education and conventional education) were provided to the participants for 8 weeks (from weeks 8 to 16). The participants were randomly assigned to undergo intensive consultation with feedback via telephone once per week or conventional education at an outpatient clinic. Information on BP and laboratory tests were collected at three time points (0, 8, and 16 weeks). BP was measured at the left arm using the auscultatory method in the sitting position after relaxing for at least 10 minutes. The participants underwent two 24-h urine collections and completed a dish frequency questionnaire (DFQ 55) at 0 and 16 weeks (17).

### Data Collection and Measurements

We divided the participants from KoGES and ESPECIAL into the younger and older groups. Women usually experience several metabolic and BP changes during the menopausal transition. Therefore, women in the younger and older groups were divided into the premenopausal and postmenopausal groups. Menopause was defined as the absence of menstruation for >3 months, and information on menopause was obtained from the questionnaire. Men of the same ages as the premenopausal women were defined as the younger group, and the remaining men were classified as the older group. Among the participants of the ESPECIAL trial, 65 underwent intensive LSD education and 65 underwent conventional LSD education in the younger group, whereas 50 underwent intensive LSD education and 59 underwent conventional LSD education in the older group. The KoGES did not collect 24-h urine data. Therefore, the amount of urine sodium per day was calculated using the INTERSALT equation (18). The urine concentration index (UCI) was calculated as the ratio of urine creatinine to serum creatinine.

### Statistical Analysis

All statistical analyses were performed using SPSS software (version 20; SPSS Inc., Chicago, IL, USA). Student's *t*-test was used to compare normally distributed variables, and data are presented as mean ± standard error of the mean. The Mann–Whitney U-test was used to compare non-normally distributed variables. Correlations were assessed using Pearson's correlation coefficients for parametric distributions. Linear regression analyses were used to determine the correlations between BP and related factors. Multiple linear regression analyses were performed to determine the correlation between urine sodium and BP after adjusting for several factors that affect BP. Statistical significance was set at *p* < 0.05.

## Results

### Pressure-Natriuresis Response Was Decreased in the Older Group in KoGES

**Figure 1:**
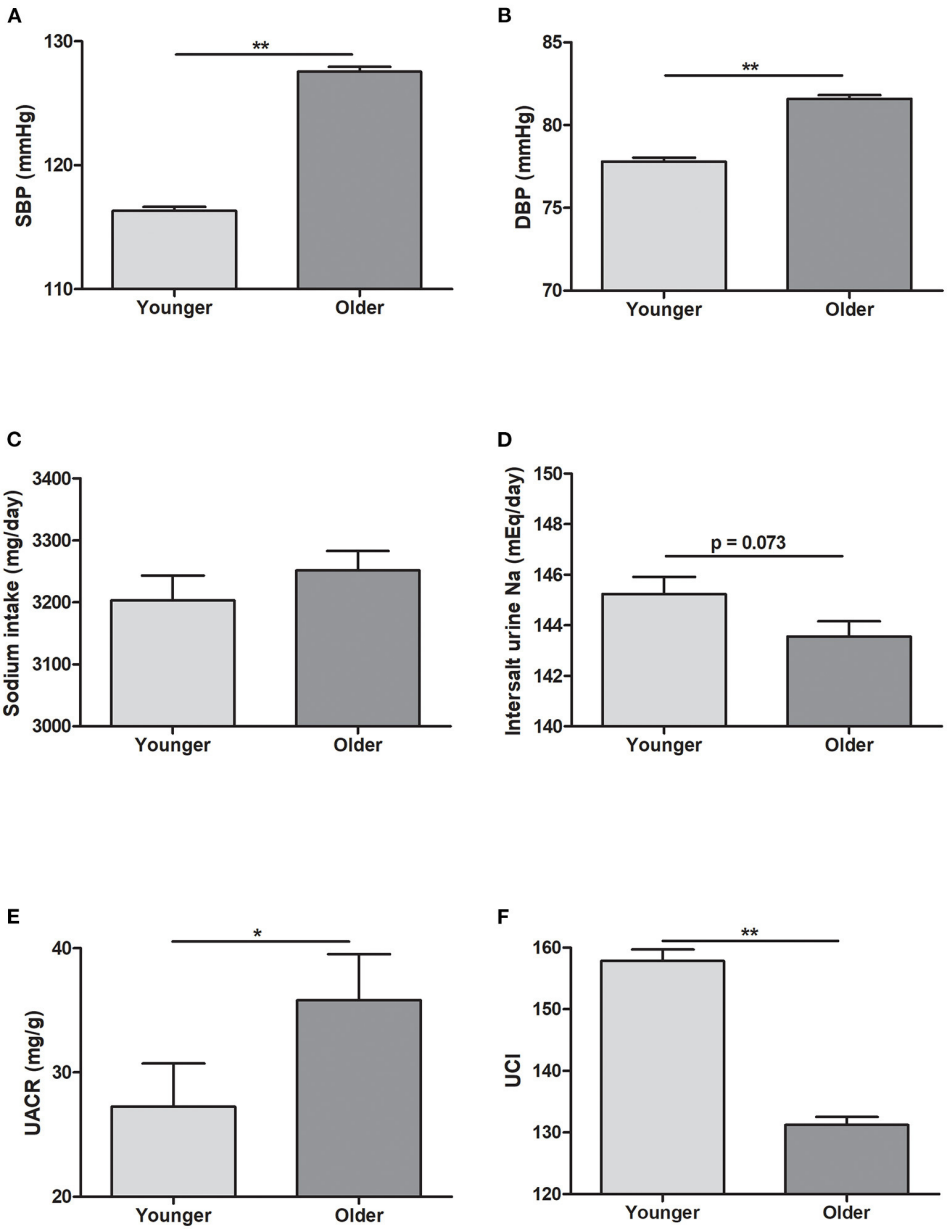
Blood pressure and urine data in the Korean Genome and Epidemiology Study (KoGES). **(A)** Systolic blood pressure (SBP). **(B)** Diastolic blood pressure (DBP). **(C)** Sodium intake (mg/day). **(D)** Sodium excretion estimated using the INTERSALT equation. **(E)** Spot urine albumin/creatinine ratio (UACR, mg/g). **(F)** Urine concentration index (UCI) in the younger and older groups. *p < 0.05 vs. different age group. **p < 0.01 vs. different age group.

**Figure 2:**
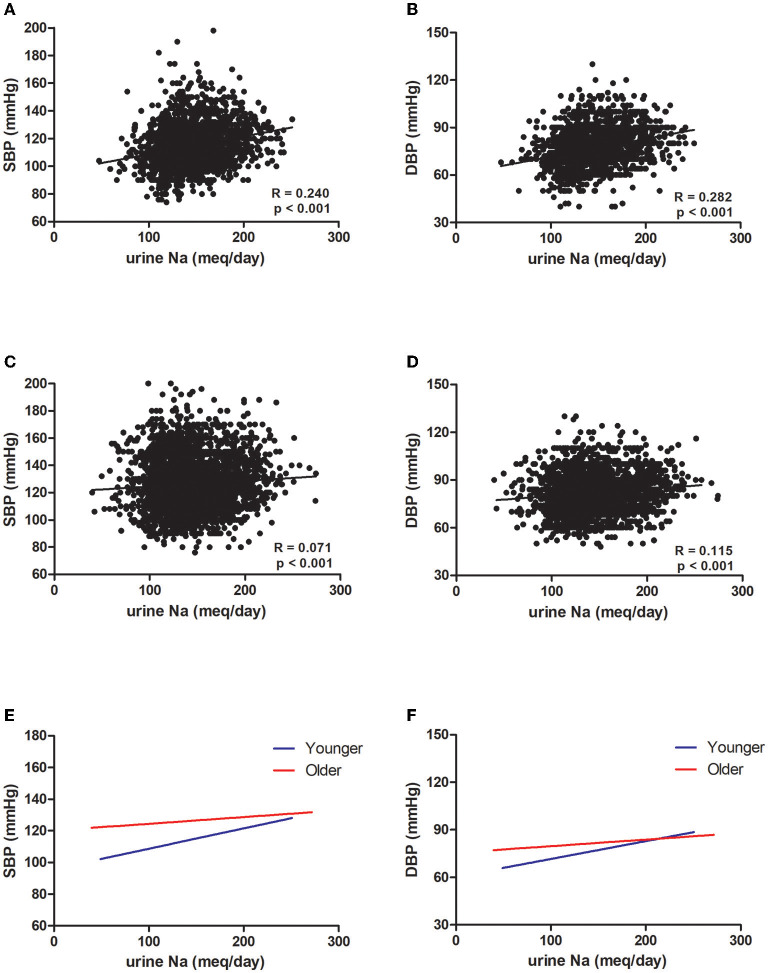
Association between blood pressure and urine sodium in the Korean Genome and Epidemiology Study (KoGES). Association of **(A)** systolic blood pressure (SBP) and **(B)** diastolic blood pressure (DBP) with urine sodium in the younger group. Association of **(C)** SBP and **(D)** DBP with urine sodium in the older group (R: correlation coefficient, *p*: *p*-value). **(E)** Linear regression of SBP and urine sodium in both groups. **(F)** Linear regression of DBP and urine sodium in both groups (blue: younger group, red: older group).

The mean age in the younger and older groups in KoGES was 43.84 and 57.88 years, respectively (Table 1). The older group showed significantly lower eGFR and higher serum sodium levels than the younger group. However, BP was higher in the older group than in the younger group (Figures 1A,B). Sodium intake did not differ between the two age groups, whereas urine sodium excretion tended to be lower in the older group (*p* = 0.073) (Figures 1C,D). The older group showed higher albuminuria and lower UCI than the younger group (Figures 1E,F). Urine sodium level and BP were positively correlated with each other in both groups (Figures 2A–D); however, the correlation coefficient was much lower in the older group. Therefore, urine sodium excretion was less excreted in the older group at a similar BP (Figures 2E,F).

**Table 1:** Baseline characteristics of participants in the KoGES and ESPECIAL trial.

	**Younger**	**Older**	***p*-Value**
**KoGES**	***n*** **=** **1,864**	***n*** **=** **2,995**	
Age	43.84 ± 2.85	57.88 ± 6.93	<0.001
Sex (M/F)	820/1,044	1,249/1,746	0.121
BMI (kg/m^2^)	24.64 ± 3.16	24.51 ± 3.22	0.163
Hb	13.50 ± 1.78	13.56 ± 1.36	0.220
BUN (mg/dL)	13.50 ± 3.29	14.50 ± 3.63	<0.001
Cr (mg/dL)	0.80 ± 0.15	0.79 ± 0.13	0.003
eGFR (mL/min/1.73 m^2^)	102.86 ± 12.46	93.70 ± 11.87	<0.001
Na (mEq/L)	141.97 ± 2.37	142.56 ± 2.24	<0.001
K (mEq/L)	4.50 ± 0.42	4.51 ± 0.45	0.167
Cl (mEq/L)	102.88 ± 2.27	102.99 ± 2.39	0.116
**ESPECIAL**	***n*** **=** **126**	***n*** **=** **109**	
Age	40.39 ± 8.30	61.19 ± 7.61	<0.001
Sex (M/F)	76/50	41/68	<0.001
BMI (kg/m^2^)	25.11 ± 4.09	25.63 ± 3.39	0.285
Hb	14.38 ± 1.63	13.35 ± 1.72	<0.001
BUN (mg/dL)	16.13 ± 5.66	18.57 ± 6.85	0.003
Cr (mg/dL)	1.13 ± 0.43	1.17 ± 0.38	0.530
eGFR (mL/min/1.73 m^2^)	73.38 ± 25.47	59.45 ± 20.02	<0.001
Na (mEq/L)	140.11 ± 1.98	141.30 ± 2.19	<0.001
K (mEq/L)	4.22 ± 0.34	4.37 ± 0.42	0.003
Cl (mEq/L)	103.67 ± 2.52	104.61 ± 4.90	0.075

*KoGES, Korean Genome and Epidemiology Study; ESPECIAL, Effects of Low Sodium Intake on the Antiproteinuric Efficacy of Olmesartan in Hypertensive Patients with Albuminuria; BMI, body mass index; Hb, hemoglobin; BUN, blood urea nitrogen; Cr, creatinine; eGFR, estimated glomerular filtration rate calculated using the Modification of Diet in Renal Disease equation; Na, sodium; K, potassium; Cl, chloride*.

### Only the Younger Group Showed a Positive Correlation Between BP and Urine Sodium in the ESPECIAL Trial

**Figure 3:**
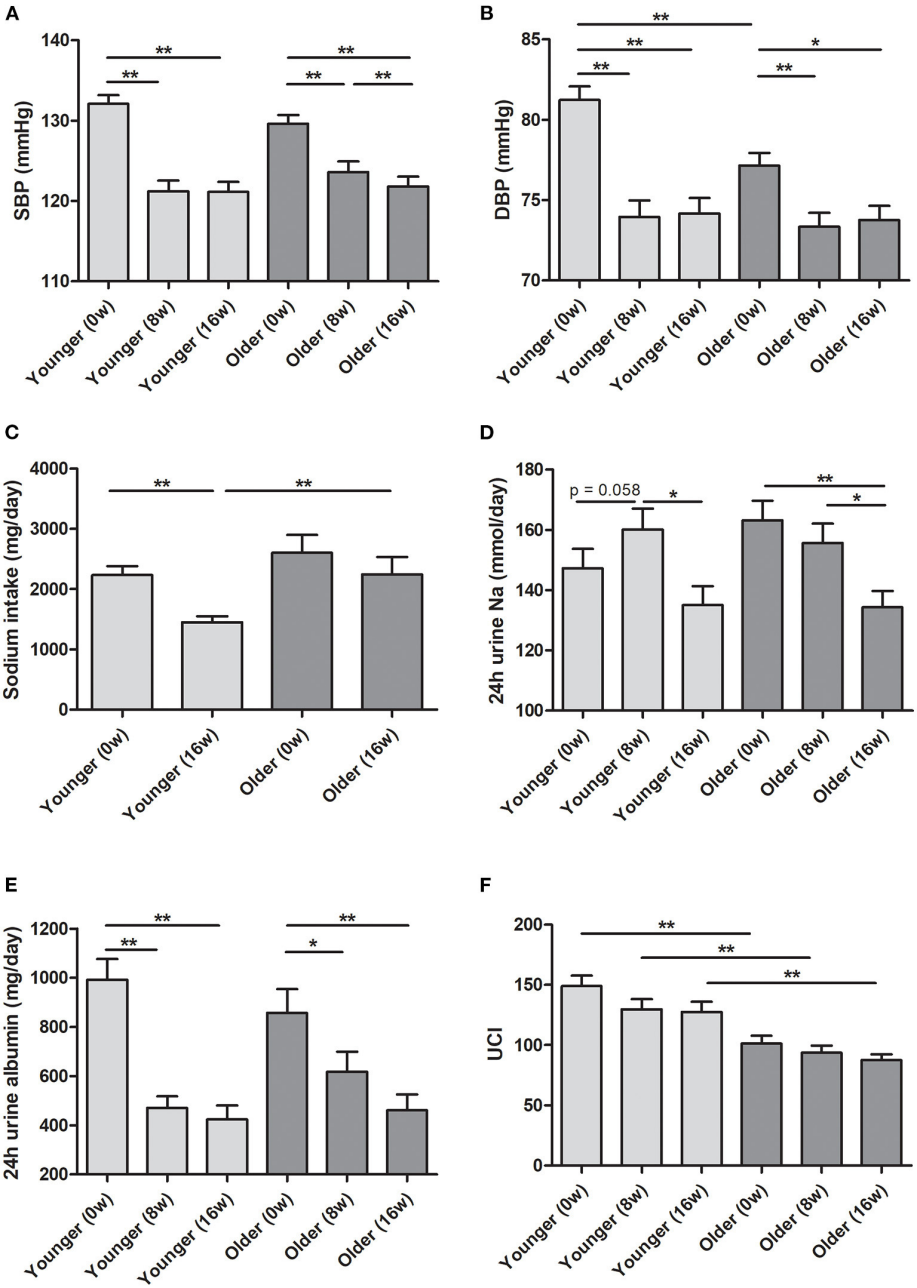
Blood pressure and urine data in the ESPECIAL (Effects of Low Sodium Intake on the Antiproteinuric Efficacy of Olmesartan in Hypertensive Patients with Albuminuria) trial. **(A)** Systolic blood pressure (SBP). **(B)** Diastolic blood pressure (DBP). **(C)** Sodium intake (mg/day). **(D)** Estimated 24-h urine sodium (mmol/day). **(E)** Estimated 24-h urine albumin (mg/day). **(F)** Urine concentration index (UCI) in the younger and older groups at 0, 8, and 16 weeks (0, 8, and 16 w, respectively). *p < 0.05 vs. different age group. **p < 0.01 vs. different age group.

**Figure 4:**
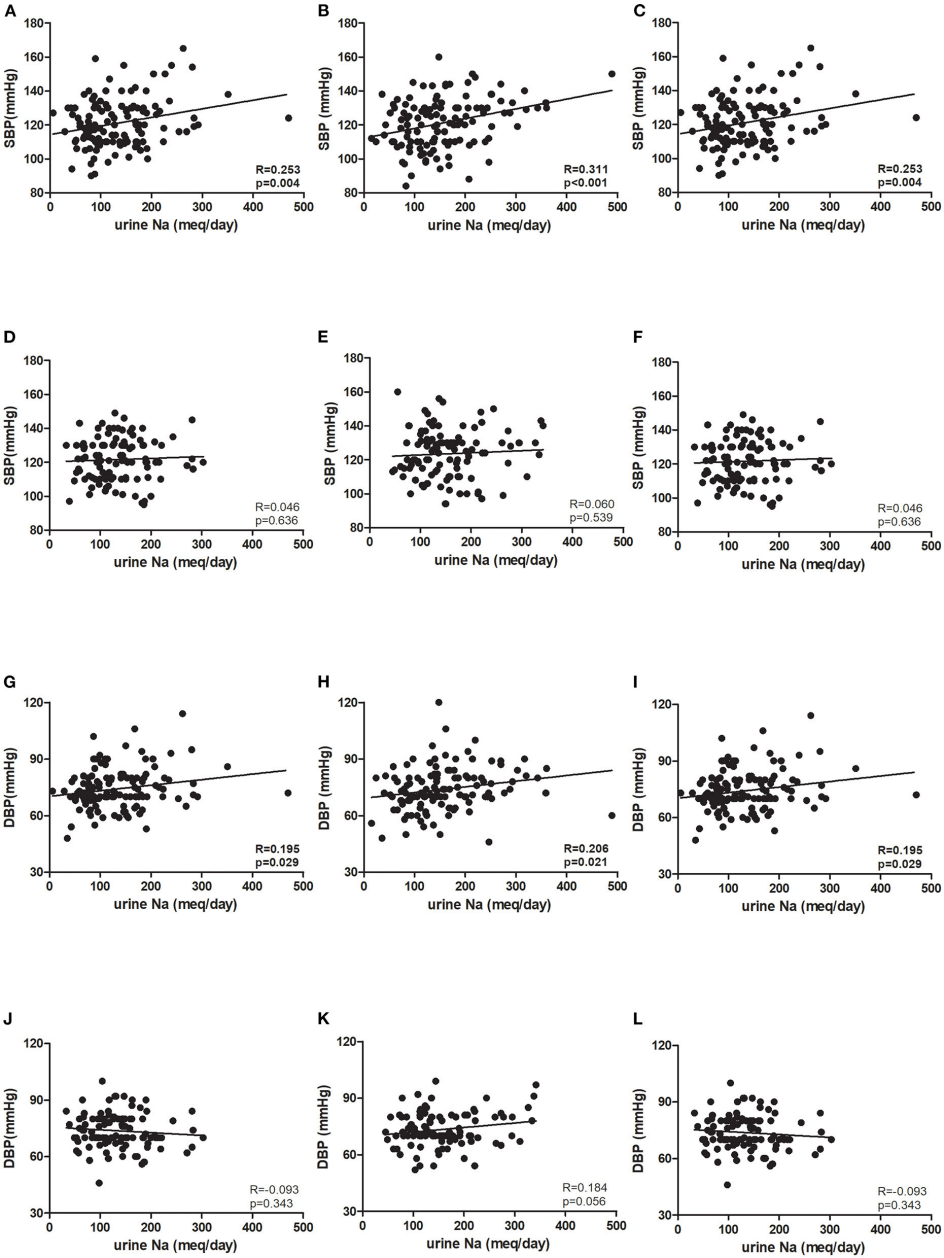
Association between blood pressure and urine sodium in the ESPECIAL (Effects of Low Sodium Intake on the Antiproteinuric Efficacy of Olmesartan in Hypertensive Patients with Albuminuria) trial. Systolic blood pressure (SBP) at **(A)** 0 week (0 w), **(B)** 8 weeks (8 w), and **(C)** 16 weeks (16 w) in the younger group. SBP at **(D)** 0 w, **(E)** 8 w, and **(F)** 16 w in the older group. Diastolic blood pressure (DBP) at **(G)** 0 w, **(H)** 8 w, and **(I)** 16w in the younger group. DBP at **(J)** 0 w, **(K)** 8 w, and **(L)** 16 w in the older group (R: correlation coefficient, *p*: *p*-value).

The mean age in the younger and older groups in the ESPECIAL trial was 40.69 and 61.19 years, respectively (Table 1). The older group included more female participants than the younger group. The older group had lower hemoglobin and eGFR, but had higher serum sodium and potassium levels. At 0 week, the systolic BP (SBP) was similar between the age groups, whereas diastolic BP (DBP) was significantly lower in the older group (Figures 3A,B). Olmesartan with or without LSD efficiently reduced BP in both groups. The baseline sodium intake was similar, whereas the sodium intake after LSD was lower in the younger group than in the older group (Figure 3C). A significant decrease in salt intake from 0 week was observed only in the younger group. Urine sodium tended to increase after olmesartan use in the younger group (*p* = 0.058), whereas it tended to decrease in the older group (Figure 3D). Albuminuria decreased with olmesartan and LSD in both groups (Figure 3E). UCI was significantly lower in the older group at all time points than in the younger group (Figure 3F). The positive correlation between urine sodium and BP was significant only in the younger group at all time points (Figures 4A–L).

### Pressure-Natriuresis Response Was Diminished in Old Age Even After Adjusting for Age, Sex, and eGFR

In KoGES, which provided participants with normal renal function, SBP and DBP were still positively associated with urine sodium excretion even after adjusting for age, sex, and eGFR (Table 2). However, the correlation coefficient was still lower in the older group. In the ESPECIAL trial, the baseline BP was not significantly correlated with urine sodium in either group. Only the younger group showed a significant positive correlation between SBP and urine sodium after olmesartan use with or without LSD in multiple regression analysis. The older group did not show any correlation between urine sodium excretion and BP.

**Table 2:** Multiple regression analysis of factors affecting blood pressure.

		**Younger**		**Older**	
		**β**	***p*-Value**	**β**	***p*-Value**
KoGES	SBP	**0.209**	**<0.001**	**0.188**	**<0.001**
	DBP	**0.206**	**<0.001**	**0.184**	**<0.001**
ESPECIAL	SBP_0 w	0.130	0.172	−0.039	0.699
	DBP_0 w	0.177	0.057	−0.018	0.854
	SBP_8 w	**0.301**	**0.002**	0.109	0.308
	DBP_8 w	0.140	0.149	0.129	0.230
	SBP_16 w	**0.258**	**0.004**	−0.005	0.960
	DBP_16 w	0.160	0.077	−0.099	0.346

*KoGES, Korean Genome and Epidemiology Study; ESPECIAL, Effects of Low Sodium Intake on the Antiproteinuric Efficacy of Olmesartan in Hypertensive Patients with Albuminuria; SBP, systolic blood pressure; DBP, diastolic blood pressure; 0 w, 0 week; 8 w, 8 weeks; 16 w, 16 weeks. Data were adjusted for age, sex, and estimated glomerular filtration rate. The bold values indicate statistically significant value*.

## Discussion

In this study, salt intake and sodium excretion did not significantly differ between the younger and older groups in the baseline. However, the pressure-natriuresis response was diminished in the older group regardless of the presence or absence of CKD. In Korean middle-aged individuals with normal renal function, salt sensitivity and BP increased in the older group. In patients with non-diabetic CKD, only younger group showed positive correlation between urine sodium excretion and SBP.

Sodium homeostasis is controlled by the integrated physiological functions of organ systems including the renal, vascular, and neurohumoral systems, which can be altered by aging. Alterations in the renal microvasculature, such as decreased numbers of glomerular and peritubular capillaries, were observed in aged Sprague–Dawley rats and aged C57Bl/6 mice and were found to be associated with increased BP and renal resistance index (19–21). Renal capillary rarefaction has been associated with decreased autoregulation of renal perfusion pressure and enhanced sodium reabsorption (22). Pasma renin activity and aldosterone concentration decreased with age, while aldosterone capacity to increase BP rather augmented with age (23). Despite the decrease of systemic RAAS, renal parenchyma ANGII was markedly elevated (24). Aging has been shown to be associated with intrarenal RAAS activation and enhanced pressor effect of ANGII infusion with decreased ANGII type 2 receptor expression and increased ANGII type 1 receptor (AT_1_R) expression in rodent models (25–27). In particular, ANGII stimulates the activity of Na+-K+-ATPase, which creates a sodium gradient that drives sodium reabsorption. Aged FBN rats on a high-salt diet were found to have markedly increased renal AT_1_R and medullary Na+-K+-ATPase protein (28). In this study, we confirmed that an ANGII receptor blocker (ARB) increased natriuresis in the younger group, whereas it failed to increase urine sodium excretion in the older group. The increased aldosterone sensitivity, intrarenal ANGII enhancement, and age-related up-regulation of sodium transporters might be the reasons for the contrasting responses to the ARB between the younger and older groups. Renal norepinephrine enhances sodium reabsorption through systemic vasoconstriction and the activation of sodium transporters (29). Aging is related to elevated sympathetic nervous system activity and enhanced renal sympathetic responsiveness (30). Several animal and human studies have demonstrated decreased renal vascular resistance, RAAS response, and renal sodium reabsorption after renal denervation (29, 31, 32). However, the specific effect of renal denervation in the older adult population has not yet been clarified.

In the ESPECIAL trial, the SBP in the older group decreased more at 8 weeks than at 16 weeks (Figure 3A). However, the SBP in the younger group did not change even with the addition of LSD to olmesartan. The effect of LSD on decreasing BP was significant only in the older group. Although our sample size was small and BP reduction was observed only in terms of SBP, some of these alterations may be associated with the age-related changes in sodium regulation. Little evidence is available on age-related changes in renal transporters. ANGII-induced Na+-K+-ATPase activity was found to be increased in the proximal renal tubules of aged FBN rats (28). In addition, an *ex vivo* study showed that increased Na+-K+-ATPase activity in proximal tubules generated a driving force to absorb apical-side sodium in aged Sabra rats (33). In distal tubules, sodium chloride cotransporters (NCCs) were expressed more in aged FBN rats (34). ANGII promoted a greater response in NCC activity in aged C57Bl6/CBA/129 mice (35). In a study in hypertensive human patients, the diuretic effect of thiazide increased with age, which can be explained by the possibility that sodium retention via NCCs may be enhanced with age (36). Owing to the reduction in arterial compliance, older adults show greater BP change regardless of changes in intravascular volume (37). In addition, older persons retain more sodium than younger persons. A randomized controlled study identified sodium restriction as a feasible strategy to decrease BP and cardiovascular events in older adults (38). In the PREMIER study, lifestyle modification including salt restriction contributed to greater BP reduction in participants aged > 50 years (39). Salt restriction is the most reliable and obvious approach to mitigate the increased salt sensitivity in older persons with hypertension. In addition, guidelines suggest that diuretics are the most effective antihypertensive drugs for older adults (40).

This study had some limitations. As 24-h urine collection was not performed in KoGES, urine sodium was estimated using the INTERSALT equation. This estimation might have reduced the accuracy of urine sodium quantification. However, urine sodium estimated with the INTERSALT equation showed better correlation with BP and sodium intake than that estimated using the Kawasaki and Tanaka equations in this study (data not shown). This study showed that the natriuretic response according to BP elevation was significantly reduced in old age by analyzing a large sample of middle-aged Koreans. In addition, this tendency did not change in older patients with non-diabetic CKD. Age-related alterations in salt regulation are a major factor contributing to BP elevation, and the role of salt restriction in achieving the target BP needs to be emphasized in the older adult population.

## Data Availability Statement

The raw data supporting the conclusions of this article will be made available by the authors, without undue reservation.

## Ethics Statement

The studies involving human participants were reviewed and approved by KHNMC 2020-01-019-002. The patients/participants provided their written informed consent to participate in this study.

## Author Contributions

HC, DK, JP, SS, BC, and CL recruited the participants. SL contributed to the design of our study and approved the final version. BO revised the paper. YK and J-YM analyzed the data and wrote the draft. All authors contributed to the article and approved the submitted version.

## Funding

This research was supported by the National Research Foundation of Korea (NRF-2020R1F1A1048586) and a grant from Kyung Hee University in 2020 (KHU-20201225).

## Conflict of Interest

The authors declare that the research was conducted in the absence of any commercial or financial relationships that could be construed as a potential conflict of interest.

## Publisher's Note

All claims expressed in this article are solely those of the authors and do not necessarily represent those of their affiliated organizations, or those of the publisher, the editors and the reviewers. Any product that may be evaluated in this article, or claim that may be made by its manufacturer, is not guaranteed or endorsed by the publisher.

## Supplementary Material

Supplementary Figure 1 Study design in the E-SPECIAL trial.
