# Supplementary figures and images for: Pressure-Natriuresis Response Is Diminished in Old Age
Source: Front Cardiovasc Med. 2022 Feb 16;9:840840. doi: 10.3389/fcvm.2022.840840 (PMC8889037; doi:10.3389/fcvm.2022.840840)

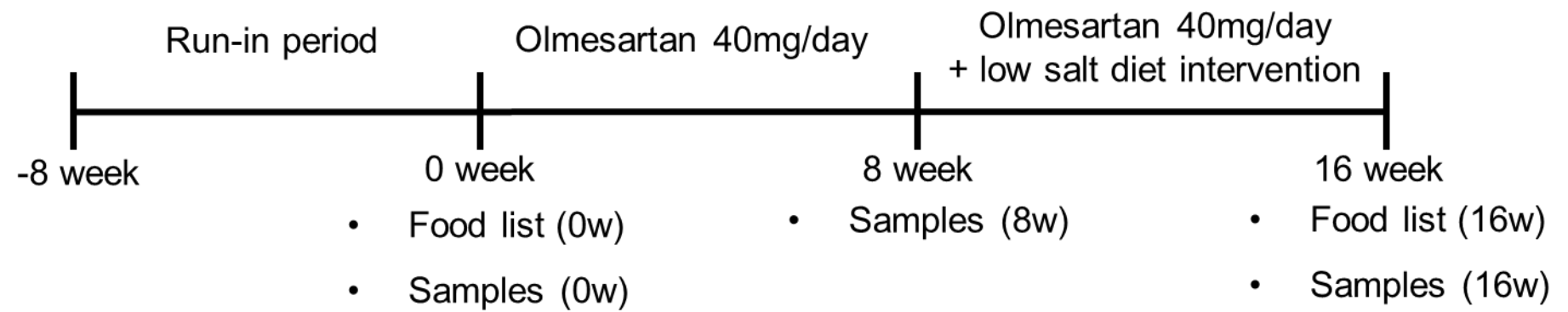

Supplement: Supplementary Figure 1 — Study design in the E-SPECIAL trial. [file Data_Sheet_1.PDF]
